# Supplementary material for: The last battle of Anne of Brittany: Solving mass grave through an interdisciplinary approach (paleopathology, biological anthropology, history, multiple isotopes and radiocarbon dating)
Source: PLoS One. 2021 May 5;16(5):e0248086. doi: 10.1371/journal.pone.0248086 (PMC8099129; doi:10.1371/journal.pone.0248086)
Supplement: S1 File — (DOCX) [file pone.0248086.s001.docx]

# Supplementary Information Text

**Title: The last battle of Anne de Bretagne: solving mass grave through an interdisciplinary approach (paleopathology, biological anthropology, history, multiple isotopes and radiocarbon dating)**

**Authors:** Rozenn Colleter^1,2,*,†,^ Clément P. Bataille^3,4,*,†^, Henri Dabernat^2^, Daniel Pichot^5^, Philippe Hamon^5^, Sylvie Duchesne^1,2^, Françoise Labaune-Jean^1,6^, Stéphane Jean^1^, Gaétan Le Cloirec^1,6^, Stefania Milano^7^, Manuel Trost^7^, Sven Steinbrenner^7^, Marine Marchal^2^, Céline Guilbeau-Frugier^8,9,10^, Norbert Telmon^2,9^, Éric Crubézy^2^, Klervia Jaouen^7,11,*,†^

# Table of Contents

[Section 1: Physical anthropology data 2](#_Toc62744808)

[Section 2: Datation 7](#_Toc62744809)

[Section 3: Lesions and paleopathological data 10](#_Toc62744810)

[Section 4: Isotopes data (δ^13^C, δ^15^N, δ^34^S, δ^18^O and ^87^Sr/^86^Sr) 19](#_Toc62744811)

[SI References 45](#_Toc62744812)

# Section 1: Physical anthropology data

## The recruitment

Twenty-nine adults and three individuals between 15 and 20 years old, all men, was counted in the two mass graves (**table S1**). The demographic profile clearly shows a significant selection of the buried persons (**fig. S1**). The group **I** included 8 individuals under 20 years of age and 34 adults (as many men as women when sex is determined) and the group **O** counted 52 adults (71% males and 29% females with a determined sex) and 11 individuals under 20 years of age (**table S1**). The Group **I** presents distortions compared to archaic mortality: (i) an over-representation of adults who died between 30 and 59 years of age and an under-represented of children under 4 years (**fig. S1**).

|  | **Number of individuals at the end of 14th – 16th century** | | | | | | | |
| --- | --- | --- | --- | --- | --- | --- | --- | --- |
| **Age/Sex** | **< 20 y.o.** | **M** | **PM** | **F** | **PF** | **Und** | **> 20 y.o.** | **total** |
| Group I | 8 | 11 | 2 | 11 | 1 | 9 | 34 | 42 |
| Group O | 11 | 29 | 0 | 12 | 0 | 11 | 52 | 63 |
| Mass Grave | 3 | 26 | 0 | 0 | 0 | 3 | 29 | 32 |
| Total | 22 | 66 | 2 | 23 | 1 | 23 | 115 | 137 |

#### **Table S1. Distribution of individuals by age group at death and by sex**. M: male, PM: probable male, F: female, PF: probable female, Und: undetermined.


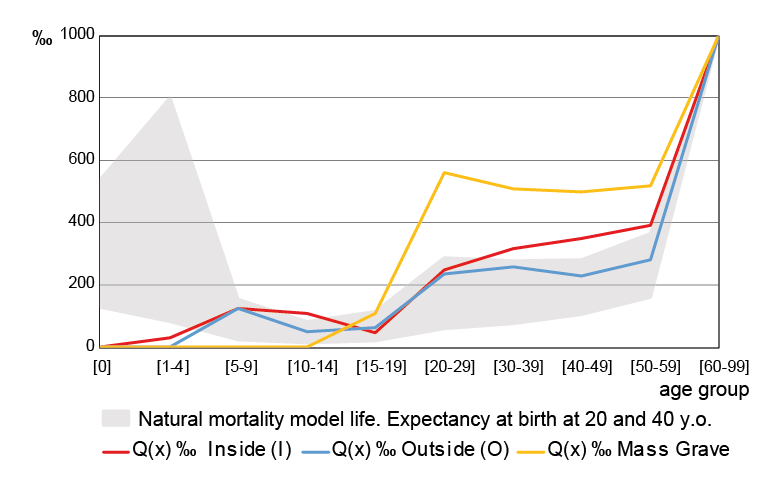


#### **Figure S1. Comparison of estimated mortality rates** (Q(x) ‰) between individual from the mass grave, other individuals buried in the convent and theoretical mortality rate for this time period. Life expectancy extremes at birth, at 20, and at 40-50 years old after Ledermann tables [1].

The statures of the individuals from the mass graves are higher than the other individuals buried in the convent (*p* = 0.00192, ANOVA, Shapiro-Wilk normality test W = 0.99108, p = 0.93) (**fig. S2A**). Those from Gr. 322 are significantly taller than those from groups **I** and **O** (**fig. S2B**); the individuals of Gr. 337 are also statistically taller than group **I**. The height standard deviation is also lower in those graves relative to other groups, which shows a certain homogeneity in morphology. If the morphology of individuals reflects both genetic and mesological parameters [2–6], the clear difference between these graves and the rest of the convent suggest a specific recruitment of the buried individuals.

Depending on the location of the tomb in the convent and the groups defined (Inside, Outside or the mass grave), the sequelae of trauma affects between 15.9 and 50% of the population studied, difference significant at *p* = 0.01582 (*χ*^2^ test = 8.2929, ddl = 3) (**fig. S7**). Only the skeletons of the mass graves have unhealed lesions and wounds in the upper part of the body (skulls and upper limbs). Trauma sequelae are present on the skeletons of 2 individuals from the Gr. 322 (skeletons 20183 and 20188) and 14 from mass Gr. 337 (**fig. S8 and table S4**). For this last tomb, it should be noted that the best represented skeletons from the southern part of the pit almost all have lesions. Five skeletons of the Gr. 337 have healed traumatic lesions (**fig. S9**) without consequence on the death of individuals 11 individuals have trauma from spiked or sharp objects, with more or less secondary lethal consequences. Among those with lethal lesions, two individuals (20764 and 20800) also show healed lesion that suggest a return to combat. For each individual in the Gr. 337 and Gr. 322, we investigated what type of lesions can be observed on their bones, as well as their location which can help to identify in what type of fight they were inflected (riders, backstabbing, etc…). Results are described in **SI Text section Lesions and paleopathological data**.

| **Id** | **Sex** | **Age group** | **Height** |
| --- | --- | --- | --- |
| **Gr. 322** |  |  |  |
| 20183 | M | [ 20 - 39 ] | 177.5 |
| 20185 | M | > 20 | 172.1 |
| 20188 | M | [ 20 - 39 ] | 166.0 |
| 20193 | M | [ 20 - 29 ] | 174.4 |
| **Gr. 337** |  |  |  |
| 20762 | M | > 20 | 162.7 |
| 20763 | M | [ 20 - 39 ] | 171.1 |
| 20764 | M | [ 20 - 29 ] | 157.5 |
| 20765 | M | [ 20 - 49 ] | 171.9 |
| 20766 | M | [ 20 - 49 ] | 169.5 |
| 20767 | M | [ 20 - 39 ] | 167.0 |
| 20768 | M | [ 20 - 29 ] | 159.9 |
| 20769 | ND | > 20 |  |
| 20776 | ND | > 20 |  |
| 20780 | ND | > 20 |  |
| 20781 | M | > 20 | 163.5 |
| 20787 | M | [ 20 - 39 ] | 164.0 |
| 20788 | M | [ 20 - 39 ] | 167.6 |
| 20789 | ND | [ 15 - 19 ] |  |
| 20790 | M | [ 20 - 49 ] | 170.5 |
| 20791 | M | [ 20 - 49 ] | 167.6 |
| 20792 | M | [ 40 - 99 ] |  |
| 20793 | ND | [ 15 - 19 ] |  |
| 20794 | ND | [ 15 - 19 ] |  |
| 20795 | M | > 20 | 164.0 |
| 20796 | M | [ 20 - 39 ] | 169.7 |
| 20797 | M | [ 20 - 39 ] |  |
| 20798 | M | [ 20 - 39 ] | 167.8 |
| 20799 | M | [ 20 - 39 ] | 174.1 |
| 20800 | M | [ 20 - 39 ] | 183.3 |
| 20801 | M | [ 20 - 39 ] | 170.0 |
| 20803 | M | [ 20 - 39 ] | 170.6 |
| 20804 | M | [ 20 - 39 ] | 161.3 |

#### **Table S2. Inventory of individuals from multiple burials with estimated age and stature**.


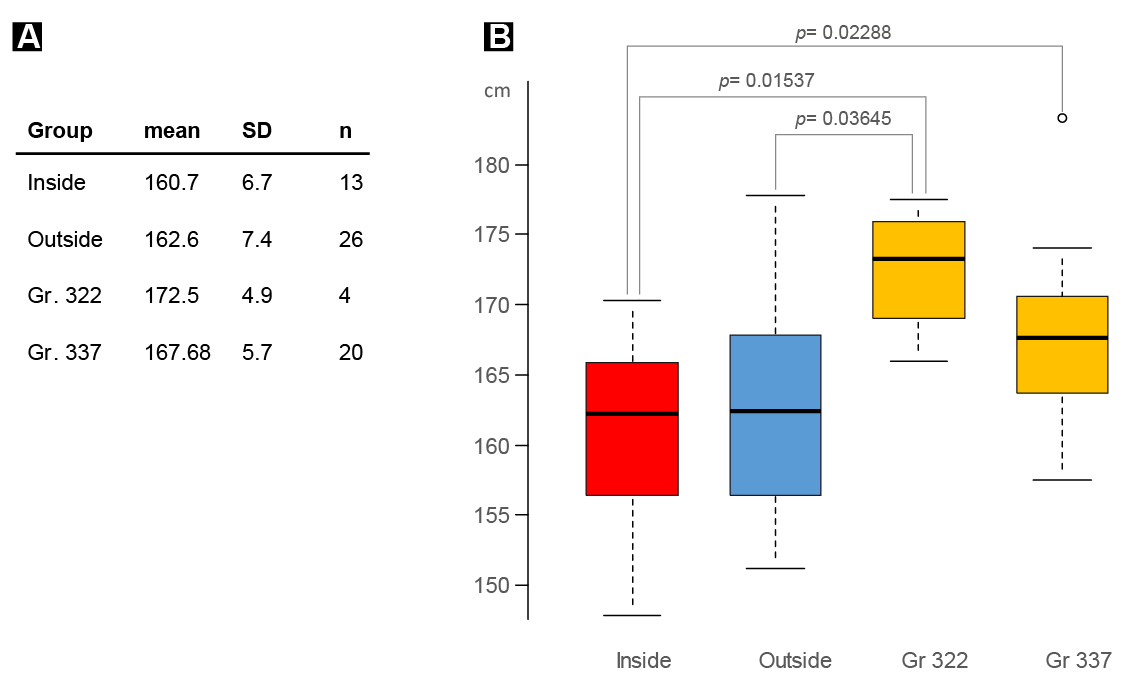


#### **Figure S2. Comparison of stature estimate between individual from the mass grave and other individuals buried in the convent.** A. Mean and standard deviations of male heights by group (SD: standard deviation, n: number of individuals); B. Whisker boxplot showing the distribution of stature value of men and significant differences between groups (Tukey tests). Tests were performed for groups classified according to their location within the convent inside (Group I), outside (Group O) or within the recovered mass graves (Mass grave).

## Funeral practices data

The individuals of these two graves were placed directly in the pits, without a coffin, contrary to the practices observed in Groups **I** and **O** (**fig. S3**). Not all individuals are systematically lying on their backs, as is almost always the case in other groups. Several individuals are placed on their right side (7/32), a position that may indicate hasty burials of the bodies. Indeed, people generally behave conservatively with their deceased in accordance with standard customs [7]. In Gr. 322, the bodies are juxtaposed and superimposed on four levels, with the first layer of individuals placed flat on their backs. However, distortions from this model appear compared to the general model as the bodies pile up: on the side, spread legs, asymmetric arms... The ones that deviate from the common positions from the outset have, on the other hand, biological characteristics that motivate these deposits, such as a stature too large for the trench.


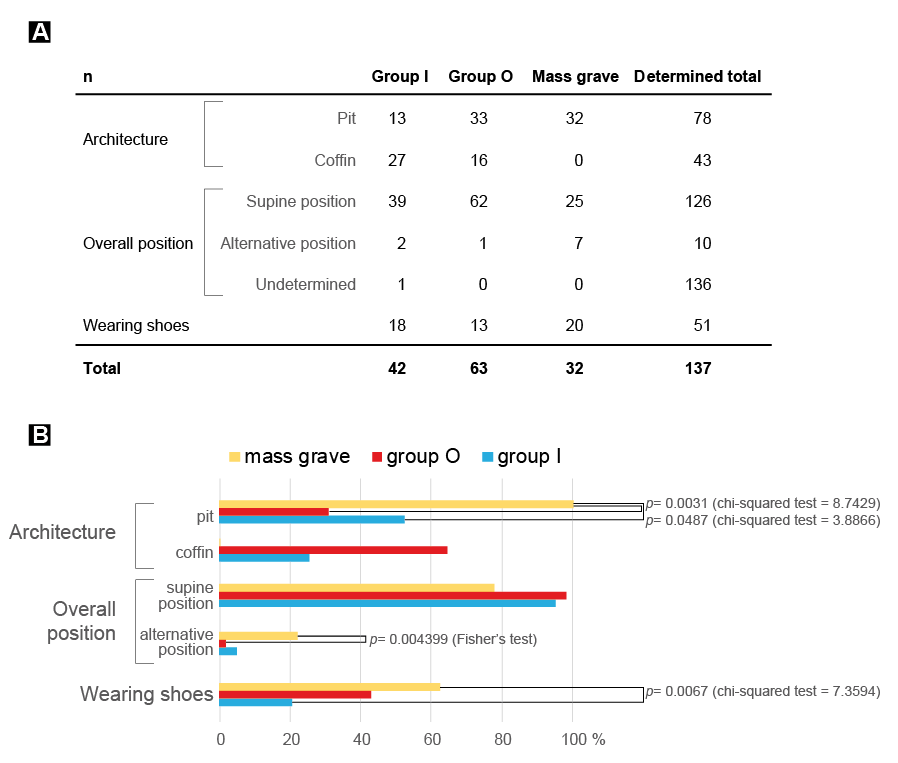


#### **Figure S3. Comparison of funeral practices between individual from the mass grave and other individuals buried in the convent.** A. Distribution of funeral practice between groups within the convent and B. Percentage of funeral practice and results of chi-squared test showing significant difference in frequency between groups. Groups are classified according to their location within the convent inside (Group I), outside (Group O) or within the recovered mass graves (Mass grave). Groups are classified according to the architecture of the tombs. Recorded funeral practice include the architecture of the grave, the general position of the bodies in the pit, and the wearing of shoes.

# Section 2: Dating

The dating of these two tombs is problematic in the absence of the discovery of objects dated *in situ* and explicit results of radiocarbon analyses carried out on the bones. Three different sets of pearls from the same jet rosary and the lower half of a bell are the only objects from the Gr. 337 (**fig. S4**). The other is totally devoid of artifacts. From the Middle Ages to the Modern Era, the shape of rosaries has undergone very little evolution and their discovery alone cannot therefore constitute a precise dating element.


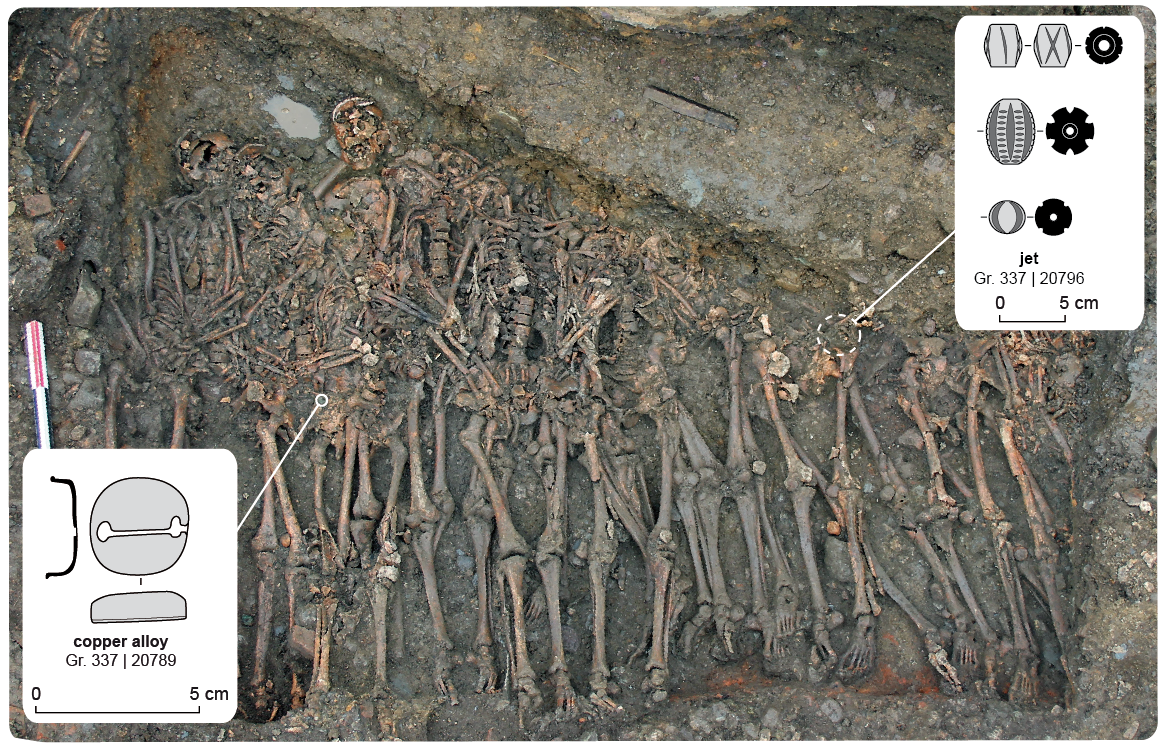
**Figure S4. Objects recovered from grave 337 and their location in the tomb**. The first-level skeletons have been removed.

In the absence of dated artefacts, we sent the samples for radiocarbon dating through Accelerator Mass Spectrometry using a selection of human remains from each tomb. The samples have been analyzed by the company Beta Analytical, Floride, USA (**fig. S5**). We used a fragment of forearm from subject 20183 (Gr. 322) and from the clavicle of 20769 (Gr. 337). The collagen was extracted with alkali by the company in 2015-2016. Several time intervals are compatible with the obtain radiocarbon values (**table S3**). The individual in grave 322 show very high probability for the interval 1436 - 1522 (76.4%) and lower probability for the interval 1574 - 1624 (19%). Individuals in grave 337 show highest probabilities for the interval 1440 - 1524 (69.3%), intermediate probabilities for the interval 1571-1630 (25.6%) and negligible probabilities for the interval 1559 - 1562 (0.5%). The lack of dating precision is due to an unfortunate plateau effect in the isotopic calibration curve between the 15th and 17th centuries.

| **Grave** | **Ind** | **Measured age** | **Corrected age *** | **Cal AD** | **Cal AD** | **δ^13^C (‰)** | **δ^15^N**  **(‰)** | **Year of analysis** |
| --- | --- | --- | --- | --- | --- | --- | --- | --- |
| 322 | 20183 | 310 +/- 30BP | 400 +/- 30BP | 1440-1520 | 1595-1620 | -19.4 | 12.0 | 2016 |
| 337 | 20769 | 300 +/- 30BP | 390 +/- 30BP | 1440-1520 | 1575-1630 | -19.4 | NA | 2015 |

#### **Table S3. Radiocarbon dates for the two graves.** Dates were calibrated using InterCal13 by Beta Analytic [8]. The age was corrected by Beta Analytical based on the δ^13^C.


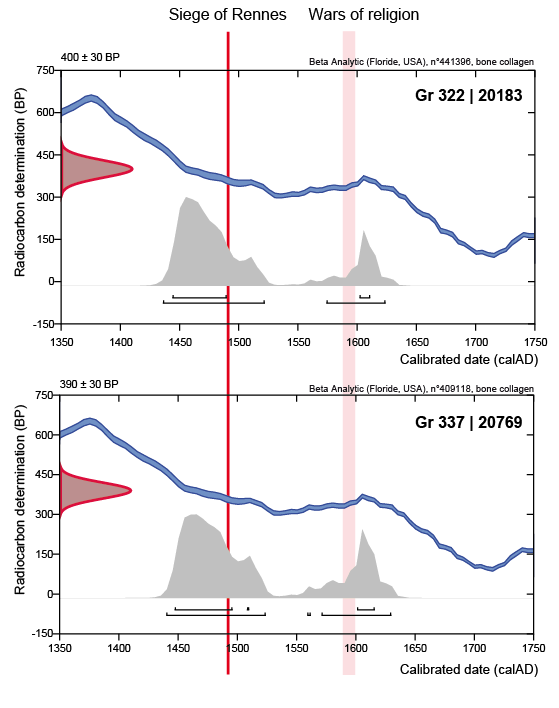


#### **Figure S5. Radiocarbon dating of human remains from the two mass graves**. Highest probability range method, Beta Analytic, Florida, USA.

The obtained dates overlap with two violent warlike episodes: the siege of the city of Rennes in 1491 (for which the probabilities are highest) and the religious wars at the end of the 16th century. Fighting episodes actually took place in Rennes during the siege of the city in 1491 [9]. Conversely, the wars reported to the League (1589-1599) are described as « *une série de petits combats, d’engagements particuliers, de surprises et de pillages de châteaux* » (« a series of small battles, skirmishes, surprises and looting of castles ») and especially concentrated in Breton Cornwall (far away from Rennes), according to the famous Breton historian, Arthur de la Borderie (mentionned by *9*).

Besides radiocarbon dates, a cross-cutting of evidences suggest that these two tombs were associated with the siege of Rennes in 1491. The absence of lead projectiles and gunshot wounds on the bones and in the pits are a strong argument for a medieval context as opposed to League wars, for these two graves [11]. The absence of any particular conflictual event in Rennes history, besides the siege of Rennes, also point towards this period to justify the burial of more than thirty individuals simultaneously. The sources of the convent also make no mention of these two graves which also suggest that this burial predates the 16th century. Convent's archives were rare before the 15th century but became more systematic from the 16th century onwards.

# Section 3: Lesions and paleopathological data

## Traumatic injuries

In this section, we describe in more details the traumatic injuries and paleopathological data recorded from each individual from the mass grave. We used traditional macroscopic methods and epifluorescence macroscopy to assess the lethality of the blows, their direction and the shape/type of the weapons (**fig. S6**). This meticulous description of each skeleton aims at reconstructing the causes of death of the individual and his life experience (previous injury, illnesses, etc.).


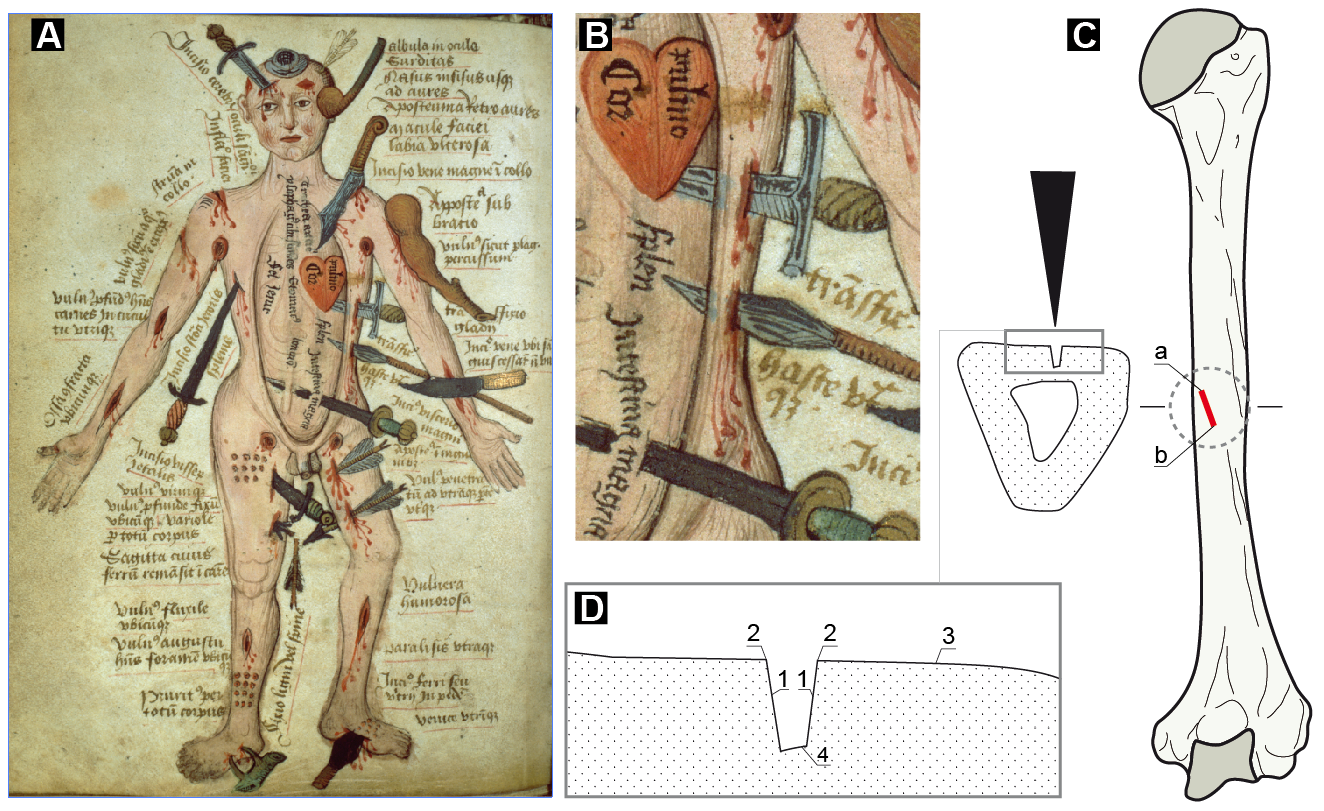


#### **Figure S6. Descriptive vocabulary of stabbing bone lesions.** A and B. The “Wound man” from an English anatomical treatise and detail of injuries (15th century) (Wound man, Pseudo-Galen, Anathomia; WMS 290. Credit: Wellcome Library, London, CC BY); C. Example of a lesion on the Humerus (a: longitudinal profile and b: transversal profile); D: Details of the bone section (1: wall; 2: bank; 3: profile and 4: bottom, deepest part of the notch).

Depending on the location of the tomb in the convent and the groups defined (Inside, Outside or the mass grave), the sequelae of trauma affects between 15.9 and 50% of the population studied, difference significant at *p* = 0.01582 (*χ*^2^ test = 8.2929, ddl = 3) (**fig. S8**).


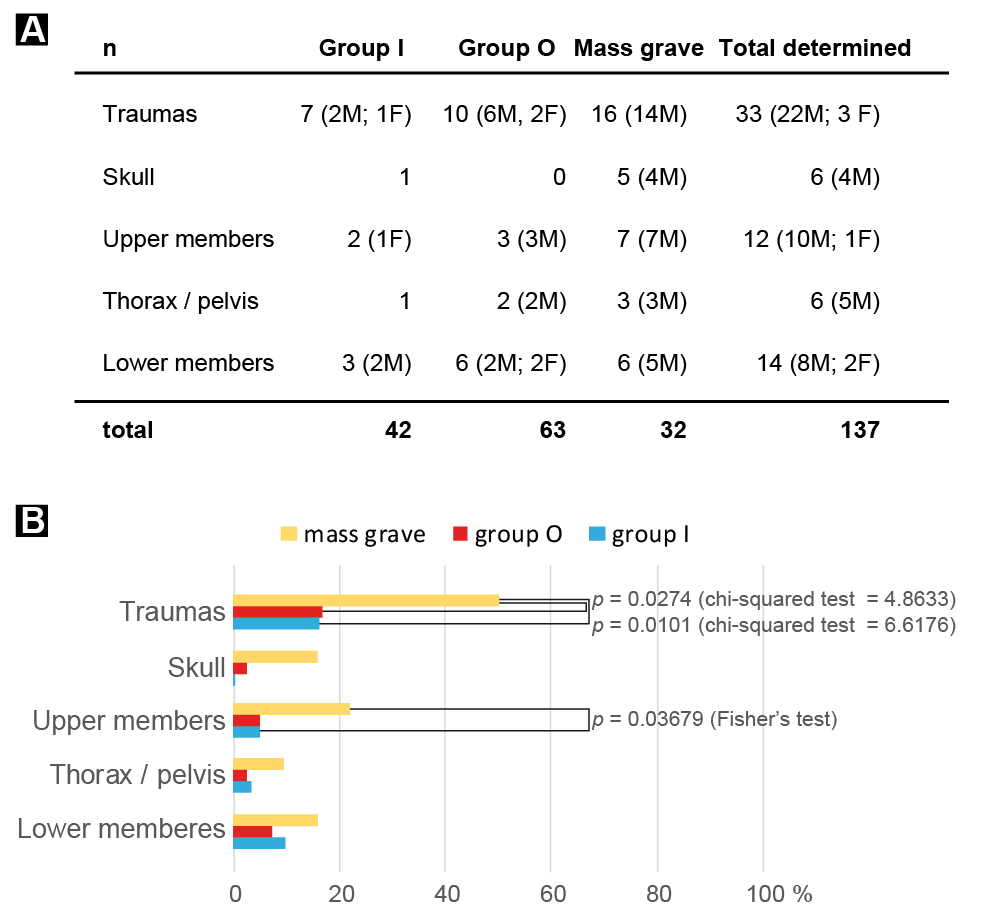


#### **Figure S7. Comparison of trauma distribution between individuals from the mass grave and other individuals buried in the convent.** **A**. Healed and unhealed trauma given as a percentage. Determined sex is given between brackets with M: male and F: female. **B.** Chi-squared tests showing significant difference in frequency between individual from the mass grave and other group buried in the convent.


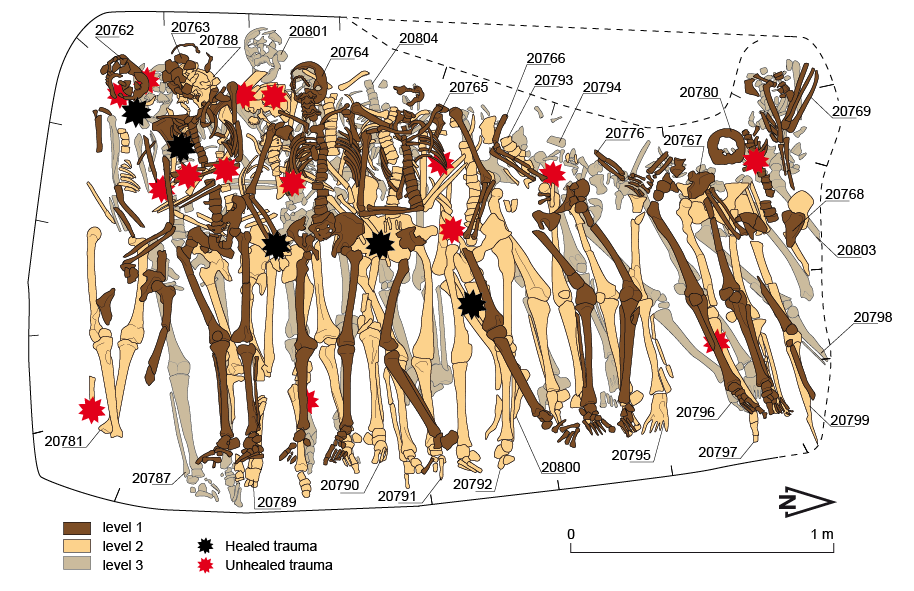


#### **Figure S8. Location of trauma carriers in tombGr.337 from level 1 (surface) to level 3 (deepest into the pit).**


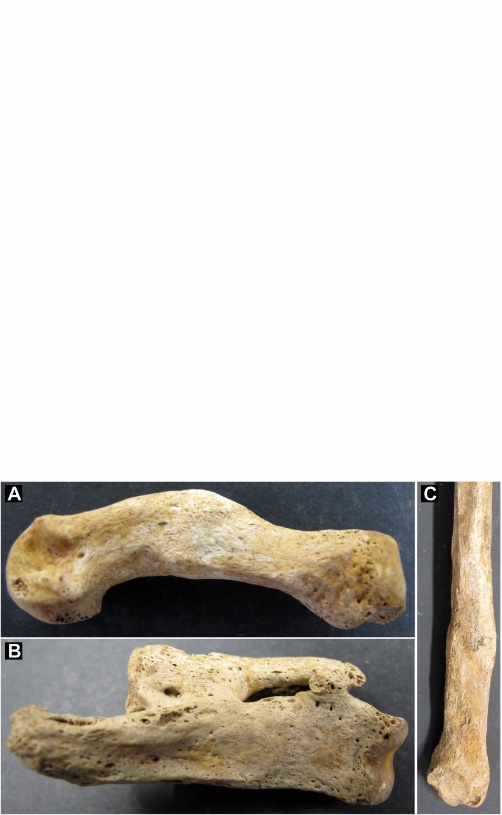


#### **Figure S9. Photos showing examples of healed trauma for Gr. 337.** A. 5th right metacarpal, lateral view, fracture with vicious callus, angulation and shortening (individual 20764); B. Right hand, row 5, phalanges without obvious fracture, dislocation with “reversal” and ankylosis (individual 20790) and C. Third distal fibula right, medial view, discrete fracture line with low angle (4-5°) and slight overlap (individual 20800).


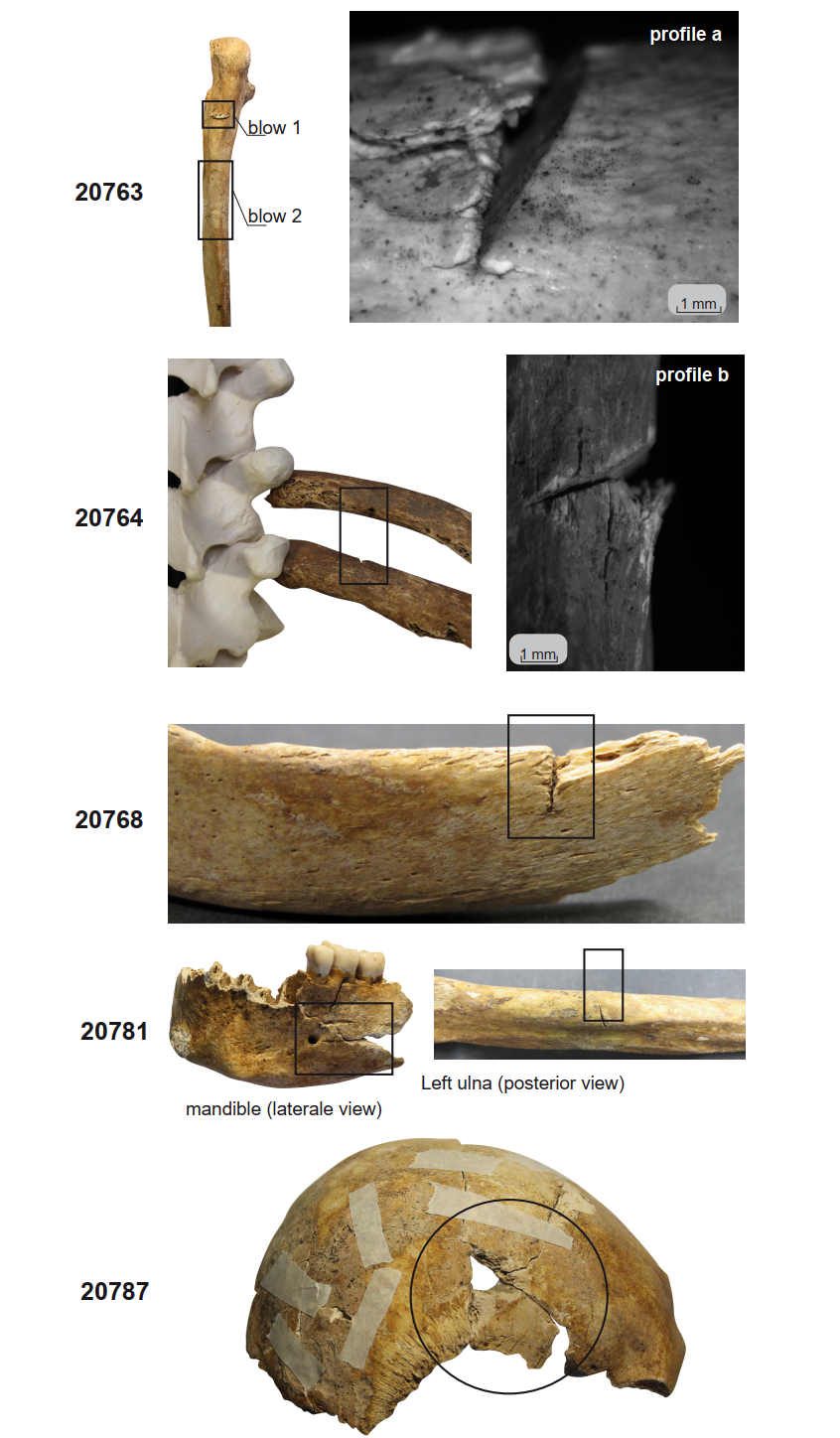


**Figure S10. Photos showing examples of blows for Gr. 337.** See Figure 3 and text for more information

**
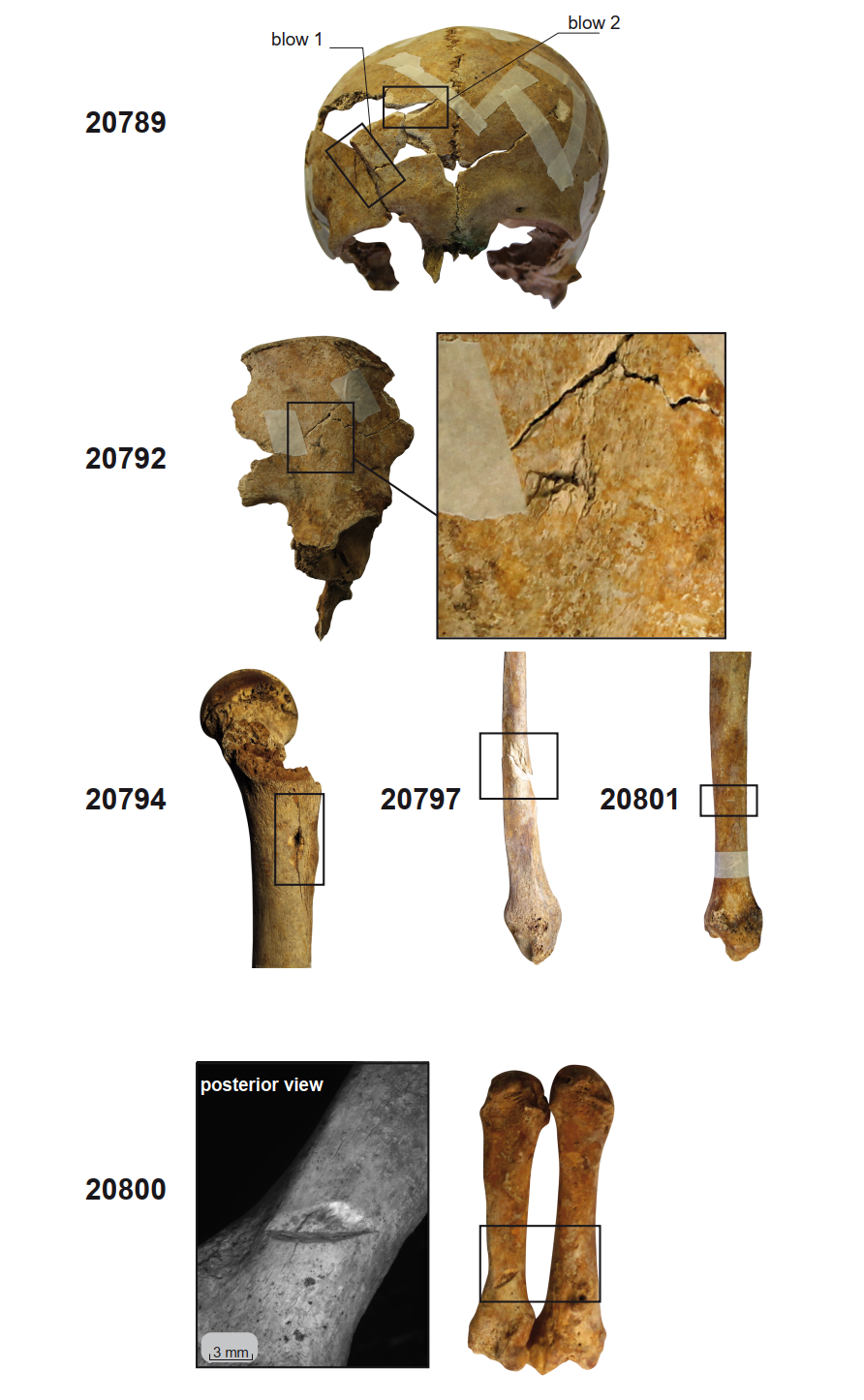
**

**Figure S11. Additional photos showing examples of blows for Gr. 337.** See Figure 3 and text for more information

|  |  |  |  | **Unhealed trauma** | | | | **Healed**  **trauma** |
| --- | --- | --- | --- | --- | --- | --- | --- | --- |
| **Id** | | **Sex** | **Age group** | **Skull** | **Upper**  **members** | **Thorax/**  **pelvis** | **Lower**  **members** |  |
| **Gr. 322** | |  |  |  |  |  |  |  |
|  | 20183 | M | [ 20 - 39 ] | 1 |  |  |  | 1 |
|  | 20185 | M | > 20 |  |  |  |  |  |
|  | 20188 | M | [ 20 - 39 ] | 1 |  |  |  |  |
|  | 20193 | M | [ 20 ; 29 ] |  |  |  |  |  |
| **Gr. 337** | |  |  |  |  |  |  |  |
|  | 20762 | M | > 20 |  |  |  |  |  |
|  | 20763 | M | [ 20 - 39 ] |  | 2 |  |  |  |
|  | 20764 | M | [ 20 - 29 ] |  |  | 1 |  | 1 |
|  | 20765 | M | [ 20 - 49 ] |  |  |  |  |  |
|  | 20766 | M | [ 20 - 49 ] |  |  |  |  |  |
|  | 20767 | M | [ 20 - 39 ] |  |  |  |  | 1 |
|  | 20768 | M | [ 20 - 29 ] |  |  | 1 |  |  |
|  | 20769 | ND | > 20 |  |  |  |  |  |
|  | 20776 | ND | > 20 |  |  |  |  |  |
|  | 20780 | ND | > 20 |  |  |  |  |  |
|  | 20781 | M | > 20 | 2 | 1 |  | 1 |  |
|  | 20787 | M | [ 20 - 39 ] | 1 |  |  |  | 1 |
|  | 20788 | M | [ 20 - 39 ] |  |  |  |  |  |
|  | 20789 | ND | [ 15 - 19 ] | 2 |  |  |  |  |
|  | 20790 | M | [ 20 - 49 ] |  |  |  |  | 2 |
|  | 20791 | M | [ 20 - 49 ] |  |  |  |  |  |
|  | 20792 | M | [ 40 - 99 ] |  |  | 1 |  |  |
|  | 20793 | ND | [ 15 - 19 ] |  |  |  |  |  |
|  | 20794 | ND | [ 15 - 19 ] |  |  |  | 1 |  |
|  | 20795 | M | > 20 |  |  |  |  |  |
|  | 20796 | M | [ 20 - 39 ] |  |  |  |  |  |
|  | 20797 | M | [ 20 - 39 ] |  |  |  | 1 |  |
|  | 20798 | M | [ 20 - 39 ] |  |  |  |  |  |
|  | 20799 | M | [ 20 - 39 ] |  |  |  |  |  |
|  | 20800 | M | [ 20 - 39 ] |  | 1 |  |  | 1 |
|  | 20801 | M | [ 20 - 39 ] |  |  |  | 1 |  |
|  | 20803 | M | [ 20 - 39 ] |  |  |  |  |  |
|  | 20804 | M | [ 20 - 39 ] |  | 1 |  |  |  |
| **Total** | |  |  | 7 | 5 | 3 | 4 | 7 |

#### **Table S4. Anatomical location and number of traumas (healed and unhealed) for individuals of the two mass graves.**

### Gr. 337

1. The skeleton 20763 is a male adult aged 20-39 years with two unhealed and uninfected impacts on the left ulna caused by sharp objects. The first is located in the upper third of the posterior surface of the diaphysis and the second, lateral under the olecranon (**Fig. 3, Fig. S10**). Injuries at the ulna may correspond to defensive injuries (parry, the victim protecting himself with his forearm in pronation). Indeed, they are not involving vessels whose section could cause significant bleeding. As a result, those injuries do not appear to have been the cause of death but may have reduced combat readiness.
2. The skeleton 20764 is that of a man aged 20-29 years with an unhealed trace of a sharp blow on the 8th and 9th right ribs (lower part) (**Fig. 3, Fig. S10**). The blow to the ribs may have caused the section of the supra-costal artery, the branch of the intercostal artery, and caused severe bleeding. The damage to the pleura resulted in pneumothorax and/or hemothorax and fatal progression. Finally, the blow could also have caused a perforation of the lung with equally serious consequences.
3. The skeleton of the young man 20767, who died between 20 and 39 years of age, has post-traumatic sequelae on his right femur (exostosis).
4. The young man who died between 20 and 29 years of age (individual 20768) is poorly represented due to a later disturbance of the head and the partial search of the left lower limb. However, sharp blow stigmas on the posterolateral faces of the 9th and 10th left ribs are observable (**Fig. 3, Fig. S10**). The blow could have caused injury to the intercostal artery, collateral to the thoracic aorta, and caused fatal bleeding by creating an hemothorax. As for individual 20764, the blow to the ribs may also have reached the pleura and lung and caused pneumothorax and/or hemothorax that could be potentially lethal as well.
5. Man 20781 is an adult over 20 years old with multiple unhealed traumas (4): left mandible hemi at the foramen, two parallel and horizontal blows; left ulna, posterior face sharp blow of 4-5 mm; tearing on the diaphysis of the right fibula (distal) (**Fig. 3, Fig. S10**). The injuries of the limbs do not show any signs of seriousness likely to be responsible for death. The one on the ulna may correspond to a parry wound, the victim protects himself with his forearm in pronation. The injury to the mandible could have been fatal.
6. The young man 20787 (died between 20 and 39 years of age) has 2 lesions on the skull: a frontal osteoma and an unhealed sharp blow to the right parietal bone. This last trauma was carried from top to bottom and from right to left and is measure 3.2 cm long (**Fig. 3, Fig. S10**).
7. Individual 20789 died between the ages of 15 and 19 and had two lethal sharp blows to the skull (**Fig. 3, Fig. S11**). The first, on the right frontal and parietal surfaces, is the result of a blow from top to bottom and from left to right (L. 9 cm). The second is also located on the right front and from top to bottom (L. preserved 2.2 cm and less than 4 cm). A bone tear 1 cm wide between blows 1 and 2 is noted on the right parietal, from top to bottom, from left to right (L. 7.2 cm).
8. The man 20790 died between the ages of 20-49. He has post-traumatic sequelae healed in his right hand: a dislocation with a phalanx reversal on the 4th metacarpal with ankylosis and bone tearing, deformity and slight angulation (fracture) of the 5th metacarpal. The left ulna has trauma sequelae on its posterior surface that may correspond to a parry fracture [12]. The presence of peripheral infection at the wound indicates that the individual survived for a time but may have reduced his combat abilities.
9. The skeleton 20792 of a man who died at over 40 years of age, is marked with a puncture wound on the right coxal (**Fig. 3, Fig. S11**). The impact of a triangular peak is noted on the hindwing of the iliac wing, not healed, from top to bottom (L. 1.5 cm). Due to the proximity of the external iliac artery, injuries to the iliac can cause significant bleeding and death.
10. The individual 20794 died between the ages of 15 and 19. He has an unhealed blow from a spiked object to the left thigh (**Fig. 3, Fig. S11**). The peak impacted the femur, on its lateral and slightly posterior surface, under the greater trochanter, from bottom to top. The transverse branch of the lateral circumflex artery may have been affected due to the proximity of its path and bone damage. The lateral circumflex artery is a collateral of the femoral artery. The blow could have caused a major bleeding since the origin of the transverse branch is very close to that of the lateral circumflex artery of the thigh.
11. The young man 20797, who died between 20-39 years of age, had an unhealed sharp blow to the right fibula (**Fig. 3, Fig S11**). It is a sharp cut on the proximal third of the bone, from bottom to top, from front to back (L. 6 mm). This injury on the limb is not of a serious nature that could be responsible for the death of the individual alone, but has certainly reduced his ability to fight.
12. The young adult male 20800 died between 20-39 years of age with a sharp blow at the base of his third left metacarpal, unhealed. Extremity injuries are not impacting the vessels and are not life-threatening (**Fig. 3, Fig. S11**). A childhood fracture is visible on his right fibula with a slight deviation.
13. The individual 20801 is a 20-39 year old adult male with unhealed sharp object trauma to the right tibia (anteromedial side) (**Fig. 3, Fig. S11**). The blow is visible on the distal third and was inflicted from top to bottom (L. 1 cm). The injuries to the extremities do not affect vessels and therefore do not appear to have been fatal, while decreasing combat skills.
14. The individual 20804 male died between 20 and 39 years of age with a mid-diaphysis bone tear on his ulna and right radius with loss of substance.

Eight of these affected bones were analyzed by epifluorescence macroscopy [13]. Three different general shapes are then described: flared in the center (individuals 20764, 20794, 20800 and 20801), arciform (individuals 20763 and 20797) and knife on the left (individuals 20768 and 20781). The general shape does not allow interpretation of the invasive agent by itself. These three shapes can be related either to the use of different weapons (of the same type or not) or to a variation in the impression of the same weapon on the bones. The length of the different lesions does not provide information on the invasive agent because the blade can slide on or penetrate the bone. It only provides descriptive information and may suggest the degree of violence of the blow. Two of the flared lesions in the center have similar widths (individuals 20764 and 20800), suggesting that the invasive agent was identical. The third lesion flared in the center (individual 20801) is wider than the two previous ones. The depth of the various lesions varies (from 180 to 4780 μm) and is sometimes difficult to obtain. It results from the strength of the attacker, which may vary according to his valor or posture. However, the depth observed on the lesion of individual 20794, although not measurable, appears to be greater than the others and suggests that the injury was caused by the tip of a perforating weapon such as the tip of a halberd. The “*rattail*”, long and out of alignment with the lesion, also shows that the weapon was hard to remove. Except the last trauma (produced by puncturing with the tip of a weapon), the injuries appear to have been caused by a sharp, blunt blade with a single thread.

The presence of this single blade yarn is determined by: (i) the flared profile, (ii) the non-parallel and non-symmetrical bank axis, (iii) the concentration of damage in the vicinity of the right bank and (iv) the presence of streaks in some straight walls (skeletons 20797, 20800 and 2080) [14]. Indeed, the thread is located on the side with the least damage. The wall-floor obliquity allows determining the orientation of the blow: half of the blows appear to have been carried obliquely (20763, 20764, 20797 and 20800) and more or less perpendicular to the bone for the other 4 (20768, 20781, 20794 and 20801). Finally, the “*rattails*” allows the determination of the direction of the blow since they mark the place where the blade left the bone. For individual 20763, the blade was removed towards the inside of the ulna (in anatomical position). For individual 20764, the blade was removed towards the outside of the rib. For individual 20794, the blade was removed along the major axis of the femoral shaft. For individual 20780, the blade was removed outwards and towards the front of the third metacarpal bone (in anatomical position). For individual 20781, the blade was removed towards the outside of the tibia (in anatomical position). The blows were made in the opposite direction to the one in which the blades were removed.

The weapons used in the 15-16th centuries include, but are not limited to, swords, sabers, halberds and other hast weapons, poniard and daggers. The invasive agent is found to be sharp by its blade and blunt by its weight. Moreover, it seems to have only one thread. Thus, in this case, saber and halberd appear to be likely candidates. The saber is a contact and cutting weapon (used to pierce and slice) that has only one thread, which distinguishes it from the sword. The halberd consists of a long wooden handle with an iron at the top of the chopper (for piercing), an axe-shaped iron for pruning (for cutting) and an iron for hooking (for knocking down the opposing rider).

### Gr. 322

It includes the proximal halves of the skeletons of four men buried simultaneously, the rest of the bodies having been dismantled from the pelvis by the installation of a wall in the courtyard (**fig. S12**). Several traces of sharp blows, some of which perimortem, were observed on two of the skeletons:

1. Individual 20183 has two blows to the frontal (fatal) and sequelae of a healed fracture on his left radius with shortening ;
2. Individual 20188 shows mandible trauma (unhealed oval perforation with no evidence of infection).


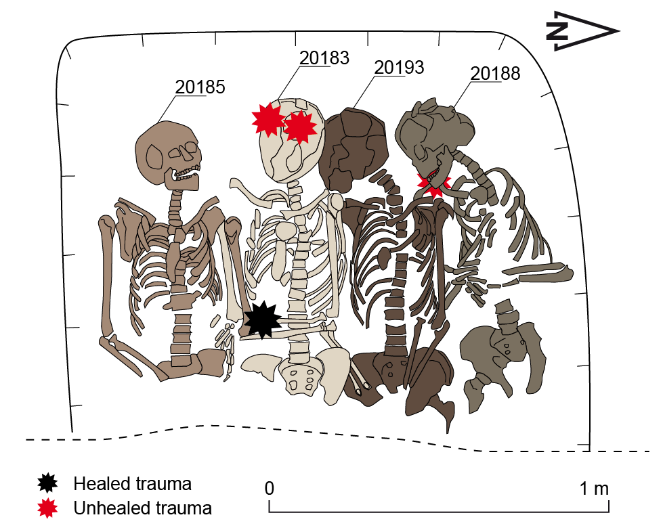


#### **Figure S12. Gr.322, location of the identified lesions.**

In summary, both mass graves are the only ones in the convent to show unhealed wounds. The general shape of the lesions is indicative of stabbing, halberd or saber type. In the Gr. 337, some shape of traumatism indicate blows from the bottom upwards, which suggests a cavalier status for these victims (individuals 20794, 20781, 20797, 20801), while other blows show the victims' completion on the ground (blows to the back). The blows to the face observed on two subjects in Gr. 322 clearly show a desire to harm the very identity of these subjects. Finally, the multiple healed traumas, whether in Gr. 332 or Gr. 337, clearly imply a return to combat (professional soldier or mercenary).

## The other damage

No evidence of infectious diseases, such as leprosy, syphilis or tuberculosis, was found on the skeletons of this series. On the other hand, non-specific infections are numerous, diagnosed in less than 25% of individuals (7/29 adults) (**table S5**). It is a rate similar to that already observed for Towton's mass grave (1461) which are linked to the risk of falls, trauma (bumps...) or surface injuries (scratches...) that large soldiers take during marches, training and battles [15].. The low levels of arthrosis observed (only 3 individuals affected from the Gr. 337) may result from a relatively low life expectancy, with an over-representation of adults under 60 years of age. Finally, the absence of stress indicators (*cribra orbitalia*) observed in the Gr. 322 (0/4 adults) contrasts with the high rates observed in the Gr. 337 (28%; 7/25 adult) and similar to Towton (more than 30% of the skulls concerned) [15] or with the medieval average which is around 10% [16]. It would mark different living conditions according to the two mass graves.

| **Group** | **Non-specific infections** | | **Arthrosis** | ***Cribra orbitalia*** |
| --- | --- | --- | --- | --- |
| **Inside** | 2 (34) | 7 | | 1 |
| **Outside** | 10 (52) | 22 | | 2 |
| **Gr. 322** | 0 (4) | 0 | | 0 |
| **Gr. 337** | 7 (25) | 3 | | 0 |
| **Total** | **19 (115)** | **32** | | **3** |

#### **Table S5. Distribution of adults by other pathology or lesions observed. Brackets: number of adults.**

# Section 4: Isotopes data (δ^13^C, δ^15^N, δ^34^S, δ^18^O and ^87^Sr/^86^Sr)

In this section, we combine several isotopic systems: carbon (δ^13^C), nitrogen (δ^15^N), sulfur (δ^34^S), oxygen (δ^18^O), and strontium (^87^Sr/^86^Sr) to obtain detailed information about the diet, geographical origin and health of the individuals recovered in the mass graves and palliate to the lack of historical record. Many of the δ^13^C, δ^15^N, and δ^34^S values were obtained in previous studies [17,18] (see below for details about the samples analyzed). We added a few analyses of δ^13^C, δ^15^N and δ^34^S values to complete the dataset for dietary reconstruction. In this work, we also present new δ^18^O and ^87^Sr/^86^Sr values for all the samples available for analysis from the two mass graves (see below for details about the samples analyzed).

## Principle of the isotope methods performed in this study: isotope systems measured

### Carbon and nitrogen isotopes: dietary indicators

Carbon and nitrogen isotopes are a common tool in archeology, and give insight into the past diet of individuals. Nitrogen isotopes are an indicator of the trophic level, whereas carbon isotopes are – in archeological European contexts - used to detect the presence of marine resources in the diet. More details can be seen in [19,20]. Carbon and nitrogen isotopes are usually analyzed by extracting the collagen of recovered bones or teeth. As the tooth roots are not renewed over the lifespan of an individual, they record the diet during the formation time of the tissue at the time of childhood depending on the growth period. The bones are renewed in 10 to 20 years and therefore record a buffered signature of the diet for approximately the last decade of life. By comparing teeth and bones, we can therefore have a glimpse into dietary changes over the lifespan of an individual between childhood and more recent life stages. For carbon isotope values, the δ^13^C values measured in collagen should mimic that of the dental enamel, though differences can often exist due to slight different integration time.

### Sulfur isotopes: mobility indicators

Sulfur isotope composition in collagen is controlled by the isotopic composition of the food consumed. Plants, at the base of food systems, have δ^34^S values influenced by the local geology and by the deposition of atmospheric sulfur from marine sulfate aerosols [21,22]. The isotope fractionation within food webs seems to be negligible [21,23]. As a result, individuals living in coastal areas, with a high deposition rate of marine sulfates, typically exhibit collagen values close to 20‰, the value of seawater, because local food systems are dominated by marine sulfate deposition. Individuals from inland areas have typically lower but more variable δ^34^S collagen values (-15 to 15‰), because the δ^34^S values of food systems are less influenced by marine sulfates and more influenced by geological controls[21–26].

### Oxygen isotopes: mobility indicators

The oxygen isotope composition (δ^18^O) of human and animal tissues that drink regularly (‘obligate drinkers’) mainly reflects the isotope composition of the local drinking water (rainfall and groundwater). Consequently, the isotopic composition measured in hard tissues (e.g., teeth, bones) can be used to predict the isotopic composition of the water consumed by individuals using equations that account for metabolic isotopic fractionation [27–29]. These equations applied are specific to the mineral fractions analyzed (carbonate and phosphate) and the species considered [27–29]. The oxygen isotope composition of the local precipitation, which control the isotopic composition of local waters, is strongly influenced by climatic and geographic factors such as temperature, latitude, altitude and distance from the coast [30–32]. These δ^18^O patterns in precipitation have been predicted at the global scale using existing isotopic data and geostatistical approaches (e.g. the Online Isotopes in Precipitation Calculator - OIPC [33]). These maps of predicting isotope patterns on the landscape (i.e. isoscape) have become a key tool in archeological studies. By comparing the isotopic signal measured in bones and teeth to the isotope range of local waters, it is possible to trace the mobility of the individuals. For this reason, this proxy has been widely used in archaeological contexts [34,35].

### Strontium isotopes

Strontium is dominantly transmitted to human tissues through ingested food [36,37]. Strontium isotope ratios (^87^Sr/^86^Sr) are strongly influenced by the local geology and do not strongly fractionate in the trophic chain [38]. The isotope ratios are influenced by the age of the bedrock, the type of bedrock, its initial content in Rb and the initial ^87^Sr/^86^Sr ratio at the time of the rock formation [39]. As rock ^87^Sr/^86^Sr is transmitted to ecosystems, many other sources of Sr with isotopically distinct ^87^Sr/^86^Sr values can influence the bioavailable ^87^Sr/^86^Sr signature [38]. For example, the addition of aerosols, mixing occurring during soil pedogenesis, differential weathering rates of minerals, anthropogenic inputs or biological processes can influence the bioavailable ^87^Sr/^86^Sr values. All those parameters cannot be easily predicted using a process-based approach, but machine-learning models have been able to predict bioavailable ^87^Sr/^86^Sr variations with sufficient precision for isotope geolocation [40,41].

All the isotope data for the samples and the standards are available in the **tables S6 to S9**.

| **Id** | **%C** | **%N** | **%S** | **C/S** | **N/S** | **C/N** | **δ^15^N** | **SD** | **δ^13^C** | **SD** | **δ^34^S** | **SD** | **ref** |
| --- | --- | --- | --- | --- | --- | --- | --- | --- | --- | --- | --- | --- | --- |
| **Grave 337** |  |  |  |  |  |  |  |  |  |  |  |  |  |
| 20797 | 39.7 | 14.7 | 0.2 | 222.7 | 82.2 | 3.2 | 10.6 | 0.1 | -19.6 | 0.0 | 5.3 |  | this study |
| 20794 |  |  |  |  |  |  | 14.6 | 0.3 | -18.0 | 0.0 | 5.4 |  | [18] |
| 20796 |  |  |  |  |  |  | 10.8 | 0.0 | -19.7 | 0.1 | 6.9 |  | [18] |
| 20800 | 42.8 | 15.7 | 0.2 | 235.8 | 86.3 | 3.2 | 11.2 | 0.0 | -19.6 | 0.1 | 7.1 |  | this study |
| 20795 | 42.8 | 15.9 | 0.2 | 212.6 | 79.1 | 3.1 | 12.2 | 0.1 | -19.4 | 0.1 | 8.3 | 0.2 | this study |
| 20787 | 39.5 | 14.5 |  |  |  | 3.2 | 11.5 | 0.1 | -19.3 | 0.2 | 8.4 |  | [18] |
| 20764 | 42.0 | 15.5 | 0.2 | 192.4 | 71.0 | 3.2 | 11.0 | 0.0 | -19.2 | 0.1 | 8.6 |  | this study |
| 20792 | 43.9 | 16.2 | 0.2 | 212.6 | 78.7 | 3.2 | 12.0 | 0.1 | -19.4 | 0.0 | 8.8 |  | this study |
| 20799 | 38.9 | 14.3 | 0.2 | 216.1 | 79.4 | 3.2 | 9.5 | 0.0 | -19.7 | 0.0 | 8.9 |  | this study |
| 20793 |  |  |  |  |  |  | 10.6 | 0.1 | -19.8 | 0.0 | 9.0 |  | [18] |
| 20791 | 44.4 | 16.4 | 0.2 | 200.9 | 74.2 | 3.2 | 12.3 | 0.1 | -19.4 | 0.1 | 9.2 |  | this study |
| 20767 | 43.4 | 16.0 | 0.2 | 205.4 | 75.9 | 3.2 | 11.9 | 0.1 | -19.4 | 0.0 | 9.3 | 0.4 | this study |
| 20769 | 40.4 | 14.9 | 0.2 | 204.3 | 75.4 | 3.2 | 11.8 | 0.2 | -19.6 | 0.1 | 9.3 |  | this study |
| 20765 | 43.0 | 15.7 | 0.2 | 202.1 | 74.5 | 3.2 | 11.2 | 0.7 | -19.4 | 0.0 | 9.4 |  | this study |
| 20790 | 44.0 | 16.3 | 0.2 | 221.3 | 82.2 | 3.1 | 12.8 | 0.1 | -19.0 | 0.0 | 9.7 |  | this study |
| 20766 | 41.6 | 15.3 | 0.2 | 184.9 | 67.9 | 3.2 | 10.7 | 0.1 | -19.8 | 0.1 | 9.8 |  | this study |
| 20803 | 39.7 | 14.7 | 0.2 | 174.7 | 64.6 | 3.2 | 12.0 | NA | -19.5 | 0.1 | 9.8 |  | this study |
| 20798 |  |  |  |  |  |  | 12.1 | 0.0 | -19.5 | 0.1 | 9.9 |  | [18] |
| 20781 | 44.1 | 16.3 | 0.2 | 183.8 | 67.9 | 3.2 | 13.0 | 0.0 | -18.8 | 0.0 | 11.8 |  | this study |
| 20789 | 42.0 | 15.5 | 0.2 | 221.3 | 82.2 | 3.2 | 12.1 | 0.2 | -19.7 | 0.0 | 12.2 |  | this study |
| 20768 | 39.7 | 14.7 | 0.2 | 195.9 | 72.7 | 3.1 | 12.2 | 0.0 | -18.1 | 0.1 | 12.6 | 0.2 | this study |
| 20801 |  |  |  |  |  | 3.3 | 9.5 | 0.0 | -20.0 | 0.1 |  |  | [18] |
| 20788 |  |  |  |  |  | 3.3 | 13.1 | 0.0 | -19.1 | 0.0 |  |  | [18] |
| **Grave 322** |  |  |  |  |  |  |  |  |  |  |  |  |  |
| 20183 | 33.9 | 12.6 | 0.2 | 158.7 | 59.1 | 3.1 | 12.8 | NA | -18.7 |  | 8.3 |  | this study |
| 20185 | 44.4 | 16.4 | 0.2 | 187.6 | 69.4 | 3.2 | 9.7 | NA | -20.1 | 0.0 | 14.9 | 0.1 | this study |
| 20188 | 39.2 | 14.7 | 0.2 | 183.1 | 68.7 | 3.1 | 12.7 | NA | -19.3 | 0.1 | 12.2 |  | this study |
| 20193 | 38.3 | 14.3 | 0.3 | 152.6 | 57.0 | 3.1 | 11.6 | 0.0 | -19.5 | 0.0 | 13.1 | 0.3 | this study |
| **Animals** |  |  |  |  |  |  |  |  |  |  |  |  |  |
| Pig |  |  |  |  |  | 3.2 | 12.7 |  | -20.2 |  |  |  | [18] |

#### **Table S6. Nitrogen, carbon and sulfur isotope values in bone collagen.** Most data were collected in this study, except for a few individuals for which the reference is provided. C/S, N/S and C/N ratios are provided to underline the good preservation of the collagen from these samples. Delta values are expressed in ‰.

| **Tooth** | | **%C** | **%N** | **%S** | **C/S** | **N/S** | **C/N** | **δ^15^N** | **SD** | **δ^13^C** | **SD** | **δ^34^S** | **SD** | **Ref** |
| --- | --- | --- | --- | --- | --- | --- | --- | --- | --- | --- | --- | --- | --- | --- |
| **Gr. 337** |  |  |  |  |  |  |  |  |  |  |  |  |  |  |
| 20787 | M3 |  |  |  |  |  | 3.3 | 11.8 | 0.04 | -19.1 | 0.04 |  |  | [18] |
| 20764 | PM3 | 41.1 | 15.2 | 0.2 | 172.7 | 63.7 | 3.2 | 11.3 | 0.4 | -19.3 | 0.20 | 8.0 |  | this study |
| 20765 | M2 | 41.0 | 15.1 | 0.2 | 178.1 | 65.3 | 3.2 | 11.5 | 0.1 | -19.3 | 0.02 | 9.8 |  | this study |
| 20781 | PM3 | 33.8 | 14.6 | 0.2 | 161.1 | 69.6 | 3.1 | 12.5 |  | -19.4 |  | 10.9 |  | this study |
| 20801 | M3 |  |  | 0.2 |  |  | 3.2 | 10.2 | 0.0 | -19.4 | 0.10 | 3.9 | 1.8 | [18] |
|  |  |  |  |  |  |  |  |  |  |  |  |  |  |  |
| **Gr. 322** |  |  |  |  |  |  |  |  |  |  |  |  |  |  |
| 20183 | PM3 | 44.7 | 16.6 | 0.2 | 198.1 | 73.3 | 3.2 | 12.9 |  | -19.1 | 0.00 | -2.4 |  | this study |
| 20185 | M2 | 45.7 | 16.9 | 0.3 | 175.6 | 64.9 | 3.2 | 9.5 |  | -20.2 | 0.03 | 14.1 |  | this study |
| 20188 | M3 | 45.6 | 17.0 | 0.2 | 187.8 | 69.9 | 3.1 | 13.0 | NA | -19.7 | 0.03 | 10.6 | 0.04 | this study |
| 20193 | M3 | 43.2 | 16.1 | 0.2 | 176.5 | 65.8 | 3.1 | 10.3 | 0.1 | -19.8 | 0.11 | 10.7 |  | this study |
|  | | | | |  |  |  |  |  |  |  |  |  |  |
| **Local individuals** | | | | |  |  |  |  |  |  |  |  |  |  |
| L. de Quengo (PM3 or PM4) | | | | |  |  | 3.3 | 14.1 |  | -19.1 |  | 14.7 |  | [18] |
| L. du Plessis (PM) | | | |  |  |  | 3.2 | 14.0 |  | -19.6 |  | 14.8 |  | [42] |
|  | | |  |  |  |  |  |  |  |  |  |  |  |  |
| **Animal** | | |  |  |  |  |  |  |  |  |  |  |  |  |
| Dog | P4 |  |  |  |  |  | 3.2 | 11.0 | 0.06 | -19.6 | 0.03 | 13.3 | 0.14 | [18] |
| Cow | M1 |  |  |  |  |  | 3.2 | 8.0 | 0.03 | -21.7 | 0.08 | 12.0 | 0.04 | [18] |
| Veal | ? |  |  |  |  |  | 3.2 | 8.6 | 0.03 | -22.0 | 0.03 | 11.4 | 0.13 | [18] |
| Sheep | ? |  |  |  |  |  | 3.2 | 9.6 | 0.02 | -21.7 | 0.04 | 12.0 | 0.57 | [18] |

#### **Table S7. Nitrogen, carbon and sulfur isotope values in tooth collagen**. Most data were collected in this study, except for a few individuals for which the reference is provided. C/S, N/S and C/N ratios are provided to underline the good preservation of the collagen from these samples. Delta values are expressed in ‰.

| **Id** | | **Tooth** | **%C** | **δ^13^C** | **δ^18^O** | **ref** | **^87^Sr/^86^Sr** | **ref** |
| --- | --- | --- | --- | --- | --- | --- | --- | --- |
| **Grave 337** | |  |  |  |  |  |  |  |
| 20787 | | M3 | 4.15 | -13.4 | -3.6 | this study | 0.71240 | [17] |
| 20764 | | PM3 | 4.10 | -15.6 | -5.9 | this study | 0.70968 | this study |
| 20765 | | M2 | 4.91 | -14.7 | -4.4 | this study | 0.70974 | this study |
| 20781 | | PM3 | 4.49 | -14.7 | -4.4 | this study | 0.70959 | this study |
| 20801 | | M3 | 5.68 | -13.3 | -6.3 | this study | 0.70900 | [17] |
| 20788 | | M3 | 3.70 | -12.9 | -4.4 | this study | 0.71092 | [17] |
| **Grave 322** | |  |  |  |  |  |  |  |
| 20183 | | PM3 | 5.10 | -14.5 | -3.9 | this study | 0.71005 | this study |
| 20185 | | M2 | 5.05 | -15.5 | -4.3 | this study | 0.71146 | this study |
| 20188 | | M3 | 5.54 | -13.8 | -3.5 | this study | 0.71084 | this study |
| 20193 | | M3 | 6.70 | -13.3 | -3.8 | this study | 0.71434 | this study |
|  | |  |  |  |  |  |  |  |
| **Local individuals** | | | | | |  |  |  |
| L. de Quengo PM3 or PM4 | | | 5.43 | -12.9 | -4.0 | [43] | 0.71079 | this study |
| L. du Plessis | PM | | 4.74 | -13.6 | -3.0 | [43] | 0.71246 | this study |
|  | | |  |  |  |  |  |  |
| **Animals** | |  |  |  |  |  |  |  |
| Dog | | P4 | 5.2 | -11.9 | -5.0 | [43] | 0.71270 | [17] |
| Pig | | p4 | 6.1 | -9.5 | -4.5 | [43] | 0.71164 | [17] |
| Cow | | M1 | 6.4 | -13.1 | -6.0 | [43] | 0.71467 | [17] |
| Veal | | ? |  |  |  | [43] | 0.71376 | [17] |
|  | | P3 | 6.4 | -11.4 | -5.0 | [43] |  | [17] |
|  | | P4 | 6.9 | -11.1 | -4.6 | [43] |  | [17] |
| Sheep | | ? |  |  |  | [43] | 0.71242 | [17] |
|  | | M1 | 6.8 | -14.2 | -3.8 | [43] |  |  |
|  | | M3 | 6.5 | -13.2 | -4.8 | [43] |  |  |

#### **Table S8. Strontium, carbon and oxygen isotope values obtained in dental enamel**. Most data were collected in this study, except for a few individuals for which the reference is provided.


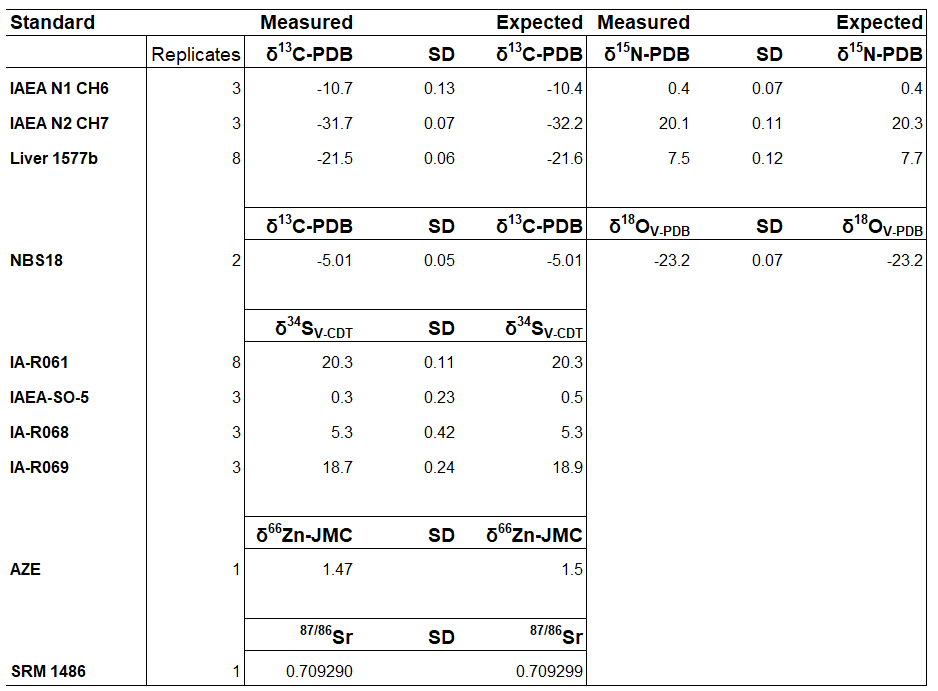


#### **Table S9. Isotope values for the different standards used in this study**.

### Additional discussion on the mass grave Gr. 322 (DNA data)

Mitochondrial DNA data for the four individuals of Gr. 322 as well as for Louise de Quengo (the local woman) have been previously published [44]. They showed that Louise de Quengo, as well as the individuals 20183, 20188 and 20193 from Gr. 322 share the same maternal haplogroup (H3-152) with a set of very specific mutations (7805A for 4 of them, with in addition 16249T for 3 of them). This has been interpreted as the sign of an endogamic group, even if the individuals were shown not to be closely related. One should remember that Louise de Quengo is born at the end of the 16th century whereas the soldiers died between the 14 and the end of the 15th centuries. The only modern individual exhibiting a H3-152 haplogroup with the 7805A mutation in the whole GenBank comes from Finistère, Brittany (individual KT799637, [45]. This genomic data strongly supports the isotope assignments and suggests that these individuals were associated with the Brittany camp. This shared haplotype and isotopic data also argue that these individuals were not mercenaries but were related by family ties to Brittany and either lived locally or came back to Brittany to help fight the war. Interestingly, the three soldiers sharing this haplotype display triple isotope geographic assignments that are not centered on Rennes, while the fourth soldier (20185, haplotype U52a) only shows values compatible with the local range, in bones and teeth. The individual 20185 is also slightly isolated from the other skeletons in the grave (**fig. S13** and **Fig. 1B**). He exhibits significantly lower δ^15^N isotope values in bones and teeth compared to the three other members of the grave, which would imply a diet poor in animal proteins (**fig. S13**). The δ^15^N values are lower than any values measured in the convent for this time (**fig. S13**) whereas the individuals 20193 and 20183 have values overlapping with the noble individuals buried in the choir and the chapels of the convent suggesting a high social status. The individual 20188 had a strong enrichment of animal protein in its diet between the childhood and adulthood, which can be related to a change of environment as seen with the δ^34^S values. Taken together, the specificity of the diet, the geographical origins, the genetic data and the position of the bodies in the grave, we argue that the individual 20185 is a local individual from a lower social status while the other individuals, 20188, 20183 and 20193, were from higher social status from an endogamic group which includes Breton individuals. The enrichment of the sulfur isotope ratios during the lifespan argues for the presence of these individuals in Brittany at least a few years before their death. It is very likely that the individuals buried there were fighting on the Breton side.


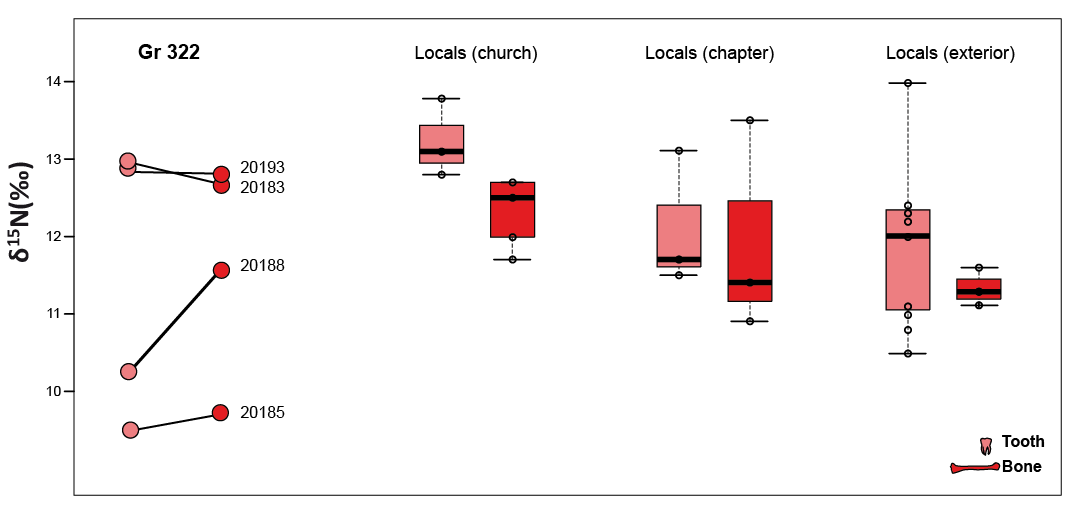


#### **Figure S13.** **Nitrogen isotope values of the teeth and bones** of the individuals from the mass grave 322 (in red) compared to local individuals analyzed in Colleter et al. [18].

# SI References

1. Ledermann S. Nouvelles tables-types de mortalité. Paris: Presses Universitaires de France; 1969.

2. Schreider E. Consanguinité et variations biologiques chez l’homme. Population (French Edition). 1976;31: 341–354. doi:10.2307/1530447

3. Marquer P. Endogamie, exogamie et variations de la stature et de l’indice céphalique dans la population béarnaise (Pyrénées-Atlantiques). Bulletins et Mémoires de la Société d’Anthropologie de Paris. 1979;6: 333–342. doi:10.3406/bmsap.1979.1969

4. Olivier G, Devigne G. Consanguinity and endogamy. Journal of Human Evolution. 1980;9: 261–268. doi:10.1016/0047-2484(80)90054-8

5. Susanne C. Living conditions and secular trend. Journal of Human Evolution. 1985;14: 357–370. doi:10.1016/S0047-2484(85)80042-7

6. Vercauteren M. Evolution séculaire au XXe siècle. Anthropologie biologique Evolution et biologie humaine. De Boeck Université; 2003. pp. 539–547.

7. Kenzler H. Post-medieval burial customs in Germany – an archaeological perspective on materiality and spatiality. Mortality. 2019;24: 123–144. doi:10.1080/13576275.2019.1585781

8. Reimer PJ, Bard E, Bayliss A, Beck JW, Blackwell PG, Ramsey CB, et al. IntCal13 and Marine13 radiocarbon age calibration curves 0–50,000 years cal BP. Radiocarbon. 2013;55: 1869–1887.

9. Hamon P. Le dernier siège de Rennes en 1491. Place Publique Rennes. 2016;39: 69–73. Available: http://www.placepublique-rennes.com/article/Le-dernier-siege-de-Rennes-en-1491

10. Croix A. La Bretagne aux XVIe et XVIIe siècles. La vie, la mort, la foi. Paris: Maloine; 1981.

11. Nicklisch N, Ramsthaler F, Meller H, Friederich S, Alt KW. The face of war: Trauma analysis of a mass grave from the Battle of Lützen (1632). PLoS ONE. 2017;12: e0178252. doi:10.1371/journal.pone.0178252

12. Judd MA. The parry problem. Journal of Archaeological Science. 2008;35: 1658–1666. doi:10.1016/j.jas.2007.11.005

13. Marchal M. Macroscopie à épifluorescence dans l’étude de lésions osseuses de coups par armes blanches sur des squelettes du XVIe siècle. Mémoire de Stage de Recherche du Master 1 Bio-Santé, Université Paul Sabatier, Toulouse III. 2015.

14. Sandras A, Guilbeau-Frugier C, Savall F, Telmon N, Capuani C. Sharp bone trauma diagnosis: a validation study using epifluorescence microscopy. Int J Legal Med. 2019;133: 521–528. doi:10.1007/s00414-018-1944-z

15. Holst MR, Sutherland TL. Towton Revisited – Analysis of the Human Remains from the Battle of Towton 1461. In: Eickhoff S, Schopper F, editors. Schlachtfeld und Massengrab: Spektren Interdisziplinärer Auswertung von Orten der Gewalt. Zossen; 2014. pp. 97–129. Available: https://www.academia.edu/10248475/Holst_M._and_Sutherland_T._2014._Towton_Revisited_Analysis_of_the_Human_Remains_from_the_Battle_of_Towton_1461_in_S._Eickhoff_and_F._Schopper_eds._Schlachtfeld_und_Massengrab_Spektren_Interdisziplin%C3%A4rer_Auswertung_von_Orten_der_Gewalt_Zossen_97-129

16. Roberts CA, Cox M. Health & Disease in Britain: From Prehistory to the Present Day. Sutton Pub.; 2003.

17. Jaouen K, Colleter R, Pietrzak A, Pons M-L, Clavel B, Telmon N, et al. Tracing intensive fish and meat consumption using Zn isotope ratios: evidence from a historical Breton population (Rennes, France). Scientific reports. 2018;8: 5077.

18. Colleter R, Clavel B, Pietrzak A, Duchesne S, Schmitt L, Richards MP, et al. Social status in late medieval and early modern Brittany: insights from stable isotope analysis. Archaeological and Anthropological Sciences. 2019;11: 823–837.

19. Schoeninger MJ, Moore K. Bone stable isotope studies in archaeology. Journal of World Prehistory. 1992;6: 247–296.

20. Reitsema LJ. Beyond diet reconstruction: stable isotope applications to human physiology, health, and nutrition. American Journal of Human Biology. 2013;25: 445–456.

21. Nehlich O. The application of sulphur isotope analyses in archaeological research: A review. Earth-Science Reviews. 2015. doi:10.1016/j.earscirev.2014.12.002

22. Tcherkez G, Tea I. 32S/34S isotope fractionation in plant sulphur metabolism. New Phytologist. 2013;200: 44–53.

23. Krajcarz MT, Krajcarz M, Drucker DG, Bocherens H. Prey-to-fox isotopic enrichment of 34S in bone collagen: Implications for palaeoecological studies. Rapid Communications in Mass Spectrometry. 2019.

24. Zazzo A, Monahan FJ, Moloney AP, Green S, Schmidt O. Sulphur isotopes in animal hair track distance to sea. Rapid Communications in Mass Spectrometry. 2011;25: 2371–2378.

25. Tanz N, Schmidt H-L. δ34S-value measurements in food origin assignments and sulfur isotope fractionations in plants and animals. Journal of agricultural and food chemistry. 2010;58: 3139–3146.

26. Richards MP, Fuller BT, Hedges RE. Sulphur isotopic variation in ancient bone collagen from Europe: implications for human palaeodiet, residence mobility, and modern pollutant studies. Earth and Planetary Science Letters. 2001;191: 185–190.

27. Hoppe KA. Correlation between the oxygen isotope ratio of North American bison teeth and local waters: Implication for paleoclimatic reconstructions. Earth and Planetary Science Letters. 2006;244: 408–417. doi:10.1016/j.epsl.2006.01.062

28. Pellegrini M, Lee-Thorp JA, Donahue RE. Exploring the variation of the δ18Op and δ18Oc relationship in enamel increments. Palaeogeography, Palaeoclimatology, Palaeoecology. 2011;310: 71–83. doi:10.1016/j.palaeo.2011.02.023

29. Chenery CA, Pashley V, Lamb AL, Sloane HJ, Evans JA. The oxygen isotope relationship between the phosphate and structural carbonate fractions of human bioapatite. Rapid Communications in Mass Spectrometry. 2012;26: 309–319. doi:10.1002/rcm.5331

30. Dansgaard W. Stable isotopes in precipitation. Tellus. 1964;16: 436–468. doi:10.3402/tellusa.v16i4.8993

31. Araguás-Araguás L, Froehlich K, Rozanski K. Deuterium and oxygen-18 isotope composition of precipitation and atmospheric moisture. Hydrological Processes. 2000. pp. 1341–1355. doi:10.1002/1099-1085(20000615)14:8<1341::AID-HYP983>3.0.CO;2-Z

32. Gonfiantini R, Roche M-A, Olivry J-C, Fontes J-C, Zuppi GM. The altitude effect on the isotopic composition of tropical rains. Chemical Geology. 2001;181: 147–167. doi:10.1016/S0009-2541(01)00279-0

33. Bowen GJ, Revenaugh J. Interpolating the isotopic composition of modern meteoric precipitation. Water Resources Research. 2003;39. doi:10.1029/2003WR002086

34. Evans JA, Chenery CA, Fitzpatrick AP. Bronze Age Childhood Migration of Individuals Near Stonehenge, Revealed by Strontium and Oxygen Isotope Tooth Enamel Analysis. Archaeometry. 2006;48: 309–321. doi:10.1111/j.1475-4754.2006.00258.x

35. Chenery C, Müldner G, Evans J, Eckardt H, Lewis M. Strontium and stable isotope evidence for diet and mobility in Roman Gloucester, UK. Journal of Archaeological Science. 2010;37: 150–163. doi:10.1016/j.jas.2009.09.025

36. Glorennec P, Lucas J-P, Mercat A-C, Roudot A-C, Le Bot B. Environmental and dietary exposure of young children to inorganic trace elements. Environment International. 2016;97: 28–36. doi:10.1016/J.ENVINT.2016.10.009

37. Lewis J, Pike AWG, Coath CD, Evershed RP. Strontium concentration, radiogenic (87Sr/86Sr) and stable (δ 88Sr) strontium isotope systematics in a controlled feeding study. STAR: Science & Technology of Archaeological Research. 2017;3: 45–57.

38. Bataille CP, Crowley BE, Wooller MJ, Bowen GJ. Advances in global bioavailable strontium isoscapes. Palaeogeography, Palaeoclimatology, Palaeoecology. 2020; 109849.

39. Bataille CP, Bowen GJ. Mapping 87Sr/86Sr variations in bedrock and water for large scale provenance studies. Chemical Geology. 2012;304: 39–52.

40. Willmes M, Bataille CP, James HF, Moffat I, McMorrow L, Kinsley L, et al. Mapping of bioavailable strontium isotope ratios in France for archaeological provenance studies. Applied Geochemistry. 2018;90: 75–86. doi:10.1016/j.apgeochem.2017.12.025

41. Bataille CP, von Holstein ICC, Laffoon JE, Willmes M, Liu XM, Davies GR. A bioavailable strontium isoscape for Western Europe: A machine learning approach. PLoS ONE. 2018;13. doi:10.1371/journal.pone.0197386

42. Jaouen K, Colleter R, Sonke JE, Laffont L, Méjean P. Manger à Rennes : l’alimentation des élites rennaises d’après les isotopes. Ce cœur qui ne battait que pour toi De sa vie provinciale à ses funérailles multiples : Louise de Quengo. Rennes: Presses Universitaires de Rennes; à paraître.

43. Bataille CP, Jaouen K, Milano S, Trost M, Steinbrenner S, Crubézy É, et al. Triple Sulfur-Oxygen-Strontium Isotopes Probabilistic Geographic Assignment of Archaeological Remains using a Novel Sulfur Isoscape of Western Europe. PLoS ONE. 2021.

44. Le Cloirec G. Rennes (Ille-et-Vilaine), Couvent des Jacobins, Du quartier antique à l’établissement dominicain. INRAP, Rapport de Fouilles; 2016.

45. Homo sapiens haplogroup H3-T152C! mitochondrion, complete genome - Nucleotide - NCBI. [cited 1 Jul 2020]. Available: https://www.ncbi.nlm.nih.gov/nuccore/KT799637.1
